# Commentary: Hernia, Mesh, and Topical Antibiotics, Especially Gentamycin: Seeking the Evidence for the Perfect Outcome…

**DOI:** 10.3389/fsurg.2017.00069

**Published:** 2017-11-27

**Authors:** Ekaterini Christina Tampaki, Athanasios Tampakis, Konstantinos Kontzoglou, Gregory Kouraklis

**Affiliations:** ^1^Second Department of Propaedeutic Surgery, Laiko General Hospital, School of Medicine, National and Kapodistrian University of Athens, Athens, Greece; ^2^Department of Visceral Surgery, University Hospital of Basel, Basel, Switzerland

**Keywords:** inguinal hernia, mesh, infection, prophylaxis, gentamycin

It was a pleasure to go through the manuscript by Hacan Kulacoglou (1) published in Frontiers in Surgery, which focuses on topical antibiotic prophylaxis use especially gentamycin in clean surgeries such as in inguinal hernia mesh reconstructions, as he brings up excellent discussion topics.

Indisputably there are benefits in using antibiotics topically rather than orally or intravenously, reducing the chances of bacterial resistance and presumably the risk of having a surgical site infection (SSI), however leading to unwanted effects development, with the most common being contact dermatitis (2).

It has been suggested that gentamycin, topically or parenterally administered (1), is effective especially against Gram-negative bacteria; however, its use as empirical monotherapy or in combination with other antibiotics in clean surgeries has not been officially comparatively evaluated. Antibiotic choice, duration, dose, dosing interval, and first dose timing in contaminated but also in clean fields appear significant and would rather depend on the antibiotic’s adverse effect profile, drug interactions, and the probability of bacterial resistance, especially given the emergence of bacterial resistance to third-generation cephalosporins, causing great concerns.

Although antibiotic prophylaxis is not mandatory in clean, elective operations, regular use of implants, accounting a 90% of inguinal hernia repairs, creates controversies (2). The European Hernia Society (3) does not suggest routine antibiotic prophylaxis. However, it is recommended where patient-related or procedure-related risks exist, as also Mazaki et al. indicated in a randomized controlled trial, showing that prophylaxis use proves to have effect on SSI prevention (4). Interestingly, mesh use in inguinal hernia reconstructive surgeries does not necessarily lead to greater wound infection risk (5). Latest data concerning wound infections regarding open mesh contrarily to non-mesh techniques used in inguinal hernia reconstructive surgeries suggest that deep infections surface rare and do not necessarily lead to mesh removal when mono- or multifilament mesh fabrics are applied along with drainage. Furthermore, the majority of RCTs are against using prophylaxis in hernia repair procedures, whereas benefits in low-risk patients seem scarce (6, 7).

According to the Environmental, Health, and Safety guidelines, prophylaxis in clean operative procedures is not required. However, risk factors presented including the patient’s age, immunosuppression status, co-morbidities, long operation duration, and drainage use are an exception (8). Further classification of the patients in low-risk and high-risk groups for SSIs seems mandatory. According to the most widely known risk indexes Study of the Efficacy of Nosocomial Infection Control and National Nosocomial Infection Surveillance evaluating the SSI rates, significant risk factors related to high SSI rates include abdominal operation processes, a more than 2-h procedure, wounds classified as dirty/infected or contaminated and patients being operated with more than three discharge diagnoses (9). Another significant issue underlined is the accurate and adequate estimation of SSI rates which can lead to significant bias and false results according to different definitions given concerning the SSIs, their time of occurrence, the patients follow-up and the study designs. The above moves the focus away from examining the most significant factors related to SSIs, which are the surgical microenvironment and the level of a country’s health service (9). By all means, an SSI rate of above or below 5% in many centers should not be objectively judged and implementations should be determined according to standard criteria.

Several surveys from England emphasize the surgeon’s preference in using antibiotic prophylaxis also in low-risk patients regarding hernia repair procedures despite the lack of adequate clinical evidence (10). The first study on aminoglycoside resistance in Upper Egypt showed that implementation of newer aminoglycosides in terms of more resistant bacteria eradication could help in mixed infections, with aminoglycosides and b-lactams acting synergistically, whereas among the regimens tested, gentamicin and amoxicillin combination has proven the least productive. They also concluded that the high resistance rates to aminoglycosides could be reduced by imposing efficient infection control policies. Among different nations, extremely high resistance to gentamicin (94.5%) in Turkey versus (32.6%) India was observed, whereas the most common resistant phenotype in Greece was that of kanamycin–neomycin (11), which may be attributed to deviations and variances in aminoglycoside treatment regimens. Resistance rates also rise varying widely between European countries. Reporting resistance rates to third-generation cephalosporins reached up between 20 and 30% in 2012 with a documented increase in aminoglycoside resistance rates (12).

The above highlights the vital importance of evaluating the surgical microenvironment and the level of a country’s health service as the most significant factors related to SSI rates. When examining antibiotic prophylaxis in clean surgeries, issues such as bacterial resistance development should be determined after taking into consideration annual updates from the European Antimicrobial Resistance Surveillance Network and reports on health cost increase per country, as such changes in resistance are always indicative of valuable trends.

Worth mentioning is that increased incidences of adverse effects have been recorded when adding aminoglycosides to b-lactams versus b-lactam monotherapy (13). Fruitful implementation of infection control measures will reduce the problem of bacterial resistance decreasing the length of hospital stay, treatment costs, and mortality rates. Therefore, as there is no system to regularly monitor antibiotic resistance or the treatment efficacy in the community, providing data that would help guide empirical antibiotic therapy also in the case of clean surgeries, clinically applying empirical combination antibiotic regimens in clean surgeries is a matter of great importance and has to be examined with caution.

## Author Contributions

ECT conceived of the idea and wrote the manuscript. AT helped to draft the manuscript. KK and GK helped to revise the manuscript. All authors read and approved the final manuscript.

## Conflict of Interest Statement

The authors declare that the research was conducted in the absence of any commercial or financial relationships that could be construed as a potential conflict of interest. The reviewer AK and handling editor declared their shared affiliation, and the handling editor states that the process nevertheless met the standards of a fair and objective review.

## Abbreviations

SSI, surgical site infection; SENIC, Study of the Efficacy of Nosocomial Infection Control; NNIS, National Nosocomial Infection Surveillance; EARS-NET, European Antimicrobial Resistance Surveillance Network; EHS, Environmental, Health, and Safety.
